# The prognostic significance of programmed cell death protein 1 and its ligand on lymphoma cells and tumor-immune cells in diffuse large B-cell lymphoma, not otherwise specified

**DOI:** 10.2478/raon-2024-0010

**Published:** 2024-02-21

**Authors:** Teja Cas Slak, Simona Miceska, Gorana Gasljevic, Lucka Boltezar, Veronika Kloboves-Prevodnik

**Affiliations:** Department of Cytopathology, Institute of Oncology Ljubljana, Ljubljana, Slovenia; Faculty of Medicine, University of Ljubljana, Ljubljana, Slovenia; Department of Pathology, Institute of Oncology Ljubljana, Ljubljana, Slovenia; Faculty of Medicine, University of Maribor, Maribor, Slovenia; Department of Medical Oncology, Institute of Oncology Ljubljana, Ljubljana, Slovenia

**Keywords:** diffuse large B-cell lymphoma, immunohistochemistry, PD-1, PD-L1, PAX5

## Abstract

**Background:**

Diffuse large B-cell lymphoma, not otherwise specified (DLBCL, NOS) is the most common type non-Hodgkin’s lymphoma, where the treatment of relapsed/refractory cases is the major challenge. Programmed cell death protein 1 (PD-1) and its ligand PD-L1 play a crucial role in the negative regulation of the immune response against the disease. The aim of the study was to analyze the expression of PD-1 and PD-L1 on lymphoma cells (LCs) and tumor-immune cells (TICs) and to investigate their correlation with outcome.

**Patients and methods:**

Samples from 283 patients diagnosed with DLBCL, NOS (both germinal center B cell like [GCB] and non-GCB subtypes) were included in the study. Expression of PD-1 and PD-L1 was determined using double immunohistochemical staining (D-IHC) for PD-1/PAX5 and PD-L1/PAX5 on tissue microarrays. LCs were highlighted by D-IHC to obtain more accurate results. Clinical data and histologic diagnoses were obtained from electronic data records. We correlated clinical characteristics, and PD-1 and PD-L1 expression on LCs and TICs with progression-free survival (PFS) and overall survival (OS).

**Results:**

Expression of PD-1 on TICs was observed in 38.4% and on LCs in 8.8% of cases, while PD-L1 was expressed on TICs in 46.8% and on LCs in 6.5% of cases. PD-L1 expression on LCs was more frequent in non-GCB subtype (p = 0.047). In addition, patients with PD-L1 expression on LCs had significantly shorter PFS (p = 0.015), and the expression retained significant in the multivariate model (p = 0.034).

**Conclusions:**

PD-L1 was more frequently expressed in LCs of the non-GCB subtype. Additionally, PD-L1 in LCs may predict shorter PFS time. D-IHC staining for PD-L1/PAX5 is a feasible method to assess PD-L1 expression on LCs of DLBCL, NOS patients and can be used to identify patients who may benefit from targeted immunotherapy with checkpoint inhibitors.

## Introduction

Diffuse large B-cell lymphoma, not otherwise specified (DLBCL, NOS) is the most prevalent type of non-Hodgkin’s lymphoma (NHL) arising from a complex interplay of genetic and molecular factors. This heterogeneity results in approximately 35% of DLBCL, NOS cases not responding to standard treatment method that combines rituximab with anthracycline-based chemotherapy. As a result, patients who do not respond to standard therapy experience relapsed or refractory disease, which remains the leading cause of mortality.^1,2^ In recent years, a variety of novel therapies, including immunotherapies, have emerged that may provide effective treatment strategies for DLBCL, NOS patients, particularly for relapsed or refractory disease. Identifying additional biomarkers and carefully assessing them to precisely define the effectiveness of immunotherapy are therefore critical for improved treatment outcomes, which are being investigated in several ongoing studies.^3,4,5^

The programmed cell death protein 1 (PD-1)/programmed cell death ligand 1 (PD-L1) immune checkpoint pathway, which is crucial for maintaining self-tolerance and excessive immune responses, has emerged as a novel biomarker target for various malignant neoplasms to suppress the anti-tumor immune response and evade immune surveillance.^6^ Immunotherapy based on the PD-1/PD-L1 signaling pathway has already been included in standard treatment guidelines for various carcinomas such as melanoma, non-small cell lung cancer, urothelial carcinoma, triple negative breast cancer and many others.^7^ Moreover, it has also attracted wide attention for the treatment of lymphomas, with the greatest success achieved in classical Hodgkin’s lymphoma and T-cell lymphoma.^8,9,10,11,12,13^ However, in DLBCL, NOS, immunotherapy based on the PD-1/PD-L1 pathway has not yet become part of the standard treatment approach. Expression of PD-1 and PD-L1 in DLBCL, NOS is often elevated, leading to impaired immune cell function and tumor growth. The expression of PD-1 has been reported mainly on immune cells, emphasizing tumor-immune cells (TICs), while PD-L1 expression on lymphoma cells (LCs) and TICs. Some studies have already shown that increased PD-1 expression on TICs is associated with favorable overall survival (OS) of DLBCL, NOS patients.^14^ In contrast, increased PD-L1 expression on LCs has been associated with poorer prognosis and increased resistance to chemotherapy^8^, which also correlates with the DLBCL, NOS non-germinal center B-cell like (non-GCB) DLBCL, NOS subtype defined by the Hans algorithm.^15^ These data confirm their pivotal role in the tumor microenvironment of DLBCL, NOS and also their contribution to the poor clinical outcomes.^8,14^ However, to date, reported data have yielded conflicting results, particularly in relation to PD-1 expression on TICs, which has been by some authors associated with better progression-free survival (PFS) and OS, while some other studies have found no association with survival.^1,6,16,17,18,19,20^ On the other hand, some findings revealed an association of PD-L1 expression on LCs with worse OS^1,6,8,14,16,18,19,20,21^, although there are data disputing the prognostic significance of PD-L1 or even showing a correlation with better rather than worse outcomes. It is also worth noting that most studies on the expression of PD-1 and PD-L1 in DLBCL, NOS have focused on the Asian population, where there is a higher prevalence of non-GCB DLBCL, NOS subtypes^22^, which has led to a lack of comprehensive research in the European population. Furthermore, almost all published studies have generally included only a very small number of DLBCL, NOS patients in their analyses.^1,18,19,23,24,25^

Moreover, the majority of published research on DLBCL, NOS has merely focused on analyzing PD-1 and PD-L1 expression on LCs or TICs, and when this has been the case, the analyses had been based on only a single immunohistochemical (IHC) staining assessment. To our knowledge, the use of markers to accurately identify LCs, such as PAX5, has rarely been reported.^14^ PAX5, a member of the paired box gene family of transcription factors, is a B cell-specific activator protein that plays an important role during B lymphopoiesis. It shows consistent expression across various stages of B-cell maturation and can be identified in the majority of B-cell neoplasms, even in cases where mature B-cell markers are not expressed.^26^ Furthermore, no studies have investigated the simultaneous expression of PD-1 and PD-L1 on both LCs and TICs and their prognostic significance.

Therefore, our aim was to evaluate the simultaneous expression of PD-1 and PD-L1 on LCs and TICs in a Slovenian cohort of DLBCL, NOS patients using double IHC staining in combination with PAX5 and to investigate their association with prognosis.

## Patients and methods

### Patients

Patients who were diagnosed with *de novo* DLBCL, NOS at the Institute of Oncology Ljubljana (IOL), Slovenia, between February 2004 and May 2018, were included in the study. All patients were older than 18 years, tested negative for HIV and underwent lymph node biopsy and histology assessment before receiving any specific oncologic treatment. In addition, all patients were treated with standard treatment (R-CHOP: rituximab-cyclophosphamide, vincristine, doxorubicin and prednisone or an R-CHOP-like protocol) and radiotherapy of residual disease if needed. Each patient has given written informed consent.

### Study design

The study was conducted retrospectively, and representative lymph node excision biopsy samples were utilized. All DLBCL, NOS patients were diagnosed and subtyped according to the Hans algorithm^27^, as was previously described^28^ (classifying DLBCL, NOS by the cell-of-origin into germinal center B-cell [GCB] and non-germinal center B-cell like [non-GCB]). Double PD-1/PAX5 and double PD-L1/PAX5 IHC staining were performed on tissue microarrays (TMAs). Results were evaluated by one experienced haematopathologist (GG), following an already published criteria.^14,16,17,25,29,30^ Clinical data were obtained from the patients’ electronic medical record and were used to calculate the correlation with the survival outcomes, as well as with PD-1 and PD-L1 expression. International Prognostic Score (IPI) was calculated for each patient.^31^ Survival analysis was based on a minimum of a 5-year patient follow-up. The study was conducted in accordance with the Declaration of Helsinki and was also approved by the Republic of Slovenia National Medical Ethics Committee (No. 0120-151/2019/4).

### Immunohistochemical staining

Double IHC staining was performed for PAX5 and PD-1 as well as for PAX5 and PD-L1 to determine PD-1 and PD-L1 expression on LCs and TICs, respectively. PAX5 nuclear staining was visualized by the presence of red chromogen in B cells, including DLBCL cells, while PD-1 and PD-L1 membranous staining was visualized by the presence of brown (diaminobenzidine, DAB) chromogen. IHC staining was performed using Benchmark XT and Benchmark Ultra automated immunostainers (Ventana Medical Systems, Inc., Tucson, AZ, USA). A detailed description of the used antibodies and IHC staining protocols are shown in Table 1.

**TABLE 1.** Description of the PAX5, PD-1 And PD-L1 antibodies and immunohistochemistry staining protocols

**Primary Ab**	**Clone**	**Vendor**	**Reaction type**	**Antigen retrieval [100°C]**	**Ab dilution**	**Ab incubation time [min]**	**IHC detection kit**
PAX5	SP34	Ventana	Nuclear	CC1 56 min	RTU	32 (37°C)	UltraView Universal Alkaline Phosphatase Red
PD-1	NAT105	Dako	Cytoplasmic, Membranous	CC1 88 min	1:200	60 (37°C)	OptiView DAB
PD-L1	SP263	Ventana	Cytoplasmic, Membranous	CC1 64 min	RTU	16 (37°C)	OptiView DAB

Ab = antibody; CC1 = cell conditioning solution 1; DAB = diaminobenzidine; IHC = immunocytochemistry; RTU = ready to use

### Evaluation of PD-1 and PD-L1 expression

TICs and LCs were defined by re-evaluating hematoxylin and eosin H&E slides and IHC slides stained for Bcl-6, CD5, CD10, CD20, and MUM1. LCs were recognized according to PAX5 expression and morphology. The expression of PD-1 and PD-L1 was assessed semi-quantitatively for both LCs and TICs, using already published cut-off values. PD-1 and PD-L1 expression on TICs was assessed in three high-powered fields (HPF), and the score was categorized in four groups: score 0 (no positive cells), score 1 (less than 10 cells), score 2 (10–30 cells) and score 3 (more than 30 cells). According to the references^16,17,25^ we considered scores 0 and 1 as negative and 2 and 3 as positive. PD-1 and PD-L1 expression on LCs was categorized into negative and positive group by using cut-off of 10% and 30%, respectively.^14,29,30^

### Statistical analysis

Descriptive statistics were used to describe the basic characteristic of the data. The median and range were calculated for the age of the patients, OS, PFS, and observation time. The Chi-square test or Fisher's exact test were used to analyze if there is a difference between PD-1 and PD-L1 expression and clinicopathological characteristics of the patients. PFS was calculated as the time from diagnosis until disease progression or death from any cause, and OS was calculated as the time from diagnosis to death from any cause. The median survival of the patients was expressed in months. Kaplan Maier with log-rank test was used to compare PFS and OS between two groups. Hazard ratio (HR) and 95% confidence interval (CI) were calculated for both univariate and multivariate analysis (Cox regression model). Parameters that proved to be significant in the univariate analysis were included in the multivariate analysis. p < 0.05 was considered significant. IBM SPSS Statistics (version 28.0.1.0, IBM, Armonk, NY, USA) was used for the analysis.

## Results

### Patients’ characteristics

The study included 283 Slovenian patients diagnosed with DLBCL, NOS. However, due to incomplete data on patients’ treatment, lost follow-up or inadequate biological material, 67 patients were excluded, resulting in a final cohort of 216 patients for subsequent analyses. The median observation time for the analyzed patients was 162 months (range 60–234 months). Clinicopathological characteristics of our patient’s cohort are presented in Table 2.

**TABLE 2.** Clinicopathological characteristics of the Slovenian patient cohort (N = 216) included in the analysis

**Age at diagnosis (years)**	
Median	64
Range	27–89
≤60	84
>60	132
**Sex, N (%)**	
Male	104 (48)
Female	112 (52)
**Ann Arbor stage, N (%)**	
I	37 (17)
II	47 (22)
III	45 (21)
IV	87 (40)
**Involvement of an extranodal organ, N (%)**	
Yes	72 (33)
No	103 (48)
No data	41 (19)
**Involvement of spleen, N (%)**	
Yes	34 (16)
No	129 (60)
No data	53 (24)
**B symptoms, N (%)**	
Yes	76 (35)
No	116 (54)
No data	24 (11)
**IPI score, N (%)**	
0, 1	63 (29.2)
2	51 (23.6)
3	50 (23.1)
4, 5	54 (24.1)
**Classification according to Hans Algorithm, N (%)**	
Non-GCB	92 (43)
GCB	124 (57)
**Survival status of the patients, N (%)**	
Alive	102 (47)
Dead	114 (53)

GCB = germinal center B-cell diffuse large B-cell lymphoma (DLBCL) subtype; IPI = International Prognostic Index; N = number; non-GCB = non-germinal center B-cell like DLBCL subtype

### The expression of PD-1 and PD-L1

**FIGURE 1.**
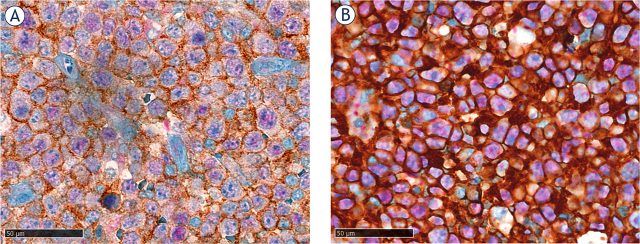
Representable images of the double immunohistochemical staining for **(A)** PD-1/PAX5 and **(B)** PD-L1/PAX5. Red chromogen indicates PAX5 in DLBCL, NOS nuclei of LCs, with brown chromogen is labeled PD-1 **(A)** or PD-L1 **(B),** respectively (40x magnification). DLBCL = diffuse large B-cell lymphoma; LCs = lymphoma cells; NOS = not otherwise specified; PD-1 = programmed cell death protein 1; PD-L1 = PD-1 ligand

PD-1 was expressed on TICs in 38.4% of cases and on LCs in 8.8% of cases, while PD-L1 was expressed on TICs in 62.5% of cases and on LCs in 6.5% of cases (Figure 1). We also investigated whether there was a difference between the non-GCB and GCB subtypes regarding PD-1 and PD-L1 expression on both TICs and LCs. Our results showed no difference in the expression of PD-1 on TICs and LCs (p = 0.291 and p = 0.224, respectively), nor for PD-L1 on TICs (p = 0.393). Interestingly, we confirmed significantly increased PD-L1 expression on LCs within the non-GCB subtype compared to the GCB subtype (p = 0.047). The detailed results of PD-1 and PD-L1 expression analysis can be found in Table 3.

**TABLE 3.** Clinicopathological characteristics of the Slovenian patient cohort (N = 216) included in the analysis

	**PD-1 on TICs Expression**		**PD-1 on LCs Expression**		**PD-L1 on TICs Expression**		**PD-L1 on LCs Expression**	
(N, %)	Positive	Negative	Positive	Negative	Positive	Negative	Positive	Negative
All cases (N = 216)	83	133	19	197	135	81	14	202
Non-GCB subtype (N = 92)	31 (37.3)	61 (45.9)	11 (57.9)	81 (41.1)	58 (43.0)	34 (42.0)	10 (71.4)	82 (40.6)
GCB subtype (N = 142)	52 (62.7)	72 (54.1)	8 (42.1)	116 (58.9)	77 (57.0)	47 (58.0)	4 (28.6)	120 (59.4)
Non-GCB versus GCB subtype (p value)	0.258		0.224		0.887		**0.047**	

GCB = germinal center B-cell DLBCL subtype; LCs = lymphoma cells; N = number; non-GCB = non-germinal center B-cell like DLBCL subtype; PD-1 = programmed cell death protein 1; PD-L1 = PD-1 ligand; TICS = tumor-immune cells

Regarding clinicopathological characteristics of the patients (Table 4), no significant differences were observed in PD-1 and PD-L1 expression on TICs or LCs when analyzed in relation with variables such as the age, sex, Ann Arbor stage, involvement of an extranodal organ, involvement of spleen, presence of B symptoms or IPI score.

**TABLE 4.** PD-1 and PD-L1 expression in association with clinicopathological characteristics of patients with diffuse large B-cell lymphoma, not otherwise specified

	**PD-1 expression on TICs**			**PD-1 expression on LCs**			**PD-L1 expression on TICs**			**PD-L1 expression on LCs**		
[N, (%)]	Positive	Negative	p value	Positive	Negative	p value	Positive	Negative	p value	Positive	Negative	p value
**Total**	83 (38.4)	133 (61.6)		19 (8.8)	197 (91.2)		135 (62.5)	81 (37.5)		14 (6.5)	202 (93.5)	
**Age**			0.775			0.466			0.885			0.406
≤60	31 (14.4)	53 (24.5)		9 (4.2)	75 (34.7)		53 (24.5)	31 (14.4)		7 (3.2)	77 (35.6)	
>60	52 (24.1)	80 (37.0)		10 (4.6)	122 (56.5)		82 (38.0)	50 (23.1)		7 (3.2)	125 (57.9)	
**Sex**			0.889			0.811			0.265			1.000
Male	39 (18.1)	65 (30.1)		10 (4.6)	94 (43.5)		61 (28.8)	43 (19.9)		7 (3.2)	97 (44.9)	
Female	44 (20.4)	68 (31.5)		9 (4.2)	103 (47.7)		74 (34.2)	38 (17.6)		7 (3.2)	1,5 (48.6)	
**Ann Arbor stage**			1.000			1.000			0.116			0.134
I–II	32 (14.8)	52 (24.1)		7 (3.2)	77 (35.6)		47 (21.8)	37 (17.1)		3 (1.4)	81 (37.5)	
III–IV	51 (23.6)	81 (37.5)		12 (5.6)	120 (55.6)		88 (40.7)	44 (20.4)		11 (5.1)	121 (56.0)	
**Involvement of an extranodal organ**			0.643			0.412			0.332			0.738
Yes	33 (18.9)	39 (22.3)		8 (4.6)	64 (36.6)		44 (25.1)	28 (16.0)		3 (1.7)	69 (39.4)	
No	43 (24.6)	60 (34.3)		7 (4.0)	96 (54.9)		71 (40.6)	32 (18.3)		6 (3.4)	97 (55.4)	
**Involvement of spleen**			0.847			1.000			0.540			1.000
Yes	15 (9.2)	19 (11.7)		3 (1.8)	31 (19.0)		25 (15.3)	9 (5.5)		1 (0.6)	33 (20.2)	
No	61 (37.4)	68 (41.7)		11 (6.7)	118 (72.4)		87 (53.4)	42 (25.8)		7 (4.3)	122 (74.8)	
**B symptoms**			0.366			0.598			0.536			0.085
Yes	27 (14.1)	49 (25.5)		5 (2.6)	71 (37.0)		52 (27.1)	24 (12.5)		9 (4.7)	67 (34.9)	
No	50 (26.0)	66 (34.4)		11 (5.7)	105 (54.7)		73 (38.0)	43 (22.4)		5 (2.6)	111 (57.8)	
**IPI score**			0.780			0.228			0.575			1.000
0–2	45 (20.8)	69 (31.9)		13 (6.0)	101 (46.8)		69 (31.9)	45 (20.8)		7 (3.2)	107 (49.5)	
3–5	38 (17.6)	64 (29.6)		6 (2.8)	96 (44.4)		66 (30.6)	36 (16.7)		7 (3.2)	95 (44.0)	
**Hans Algorithm classification**			0.258			0.224			0.887			**0.047**
Non-GCB	31 (14.4)	61 (28.2)		11 (5.1)	88 (37.5)		58 (26.9)	34 (15.7)		10 (4.6)	82 (38.0)	
GCB	52 (24.1)	72 (33.3)		8 (3.7)	116 (53.7)		77 (35.6)	47 (21.8))		4 (1.9)	120 (55.6)	
**Patients’ outcome**			0.124			0.639			0.779			0.788
Alive	45 (20.8)	57 (26.4)		10 (4.6)	92 (42.6)		65 (30.1)	37 (17.1)		6 (2.8)	96 (44.4)	
Dead	38 (17.6)	76 (35.2)		9 (4.2)	105 (48.6)		70 (32.4)	44 (20.4)		8 (3.7)	106 (49.1)	

GCB = germinal center B-cell DLBCL subtype; IPI = International Prognostic Index; LCs = lymphoma cells; N = number; non-GCB = non-germinal center B-cell like DLBCL subtype; PD-1 = programmed cell death protein 1: PD-L1 = PD-1 ligand; TICs = tumor-immune cells; % = percentage

### Clinicopathological characteristics and correlation with progression free-survival and overall survival

**FIGURE 2.**
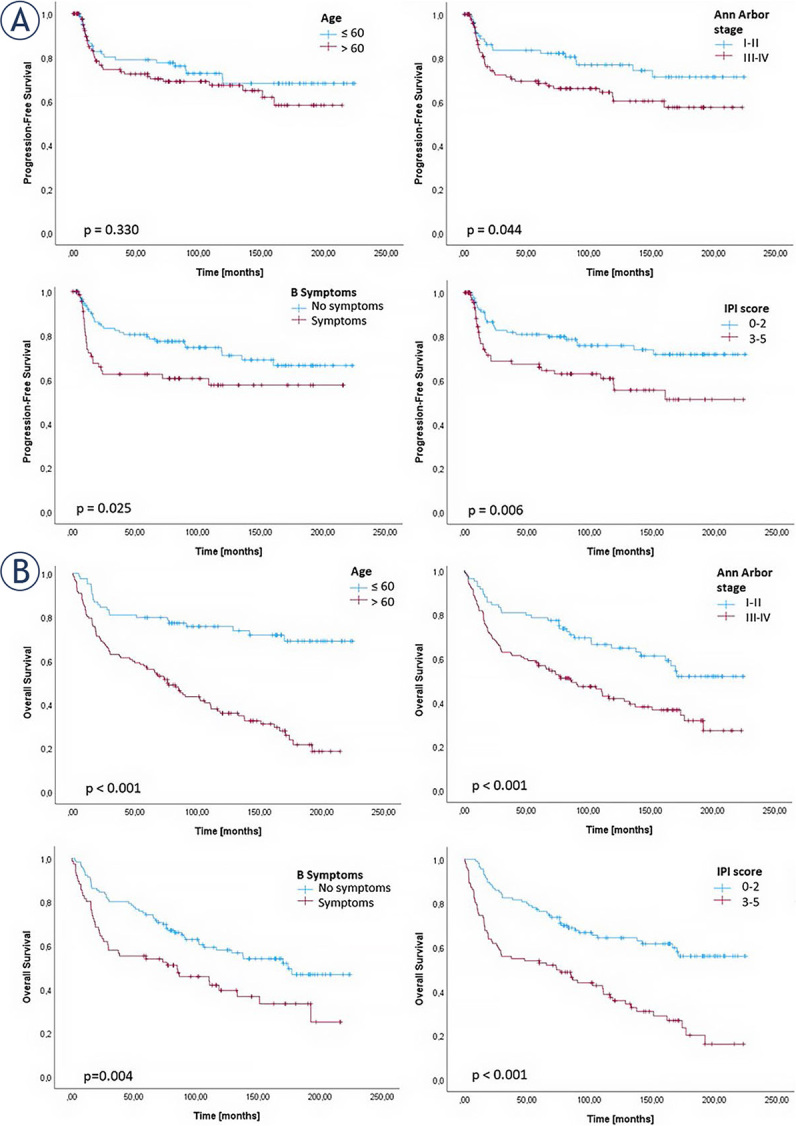
Kaplan-Meier curves for **(A)** progression-free survival and **(B)** overall survival, representing only significant differences among all analyzed clinicopathological characteristics of diffuse large B-cell lymphoma, not otherwise specified patients.

The results of the survival analysis, which was performed on the basis of the clinicopathological characteristics of the patients, are summarized in Figure 2 and Table 5. The median PFS was 77.4 months (range 0.23–224.89) and the median OS was 83.3 months (range 0.23–224.89). In the group of patients under 60 years of age, the OS was significantly longer than in the group of patients over 60 years of age (p < 0.001). At the same time, we did not find age to be statistically significant for PFS. Moreover, patients in Ann Arbor stages I or II showed a significant association with longer PFS (p = 0.044) and OS (p < 0.001) compared to patients in stages III and IV. In addition, a longer PFS (p = 0.025) and OS (p = 0.004) were observed in patients without B-symptoms compared to patients with B-symptoms. A low IPI score (score between 0 and 2) was associated with a longer PFS (p = 0.003) and a longer OS (p < 0.001). No correlation with PFS and OS was observed for the other clinicopathological characteristics such as Hans algorithm, gender, an extranodal organ and spleen involvement.

**TABLE 5.** Univariate and multivariate analysis of the patients’ survival based on their clinicopathological characteristics and PD-1 and PD-L1 expressions on lymphoma cells and tumor-infiltrating immune cells in tissue samples of diffuse large B-cell lymphoma, not otherwise specified

		**Univariate analysis**				**Multivariate analysis**			

		**PFS**		**OS**		**PFS**		**OS**	

		**p value**	**Median when patients have relapse [months]**	**p value**	**Median when patients died [months]**	**p value**	**HR (95% CI)**	**p value**	**HR (95% CI)**
**Age**	≤ 60 *vs.* > 60	0.330	91.8 *vs.* 59.7	**< 0.001**	110.1 *vs.* 73.5			**< 0.001**	2.907 (1.710–4.940)
**Sex**	Male *vs.* Female	0.945	69.1 *vs.* 80.9	0.324	78.3 *vs.* 90.8				
**Ann Arbor stage**	I–II *vs.* III–IV	**0.044**	91.3 *vs.* 59.7	**< 0.001**	113.8 *vs.* 72.0	0.845	1.072 (0.532–2.130)	0.073	1.654 (0.955–2.865)
**Involvement of an extranodal organ**	(−) *vs.* (+)	0.886	77.4 *vs.* 74.1	0.451	81.8 *vs.* 82.3				
**Involvement of the spleen**	(−) *vs.* (+)	0.915	69.9 *vs.* 81.8	0.844	80.1 *vs.* 81.8				
**B symptoms**	(−) *vs.* (+)	**0.025**	85.3 *vs.* 30.8	**0.004**	91.8 *vs.* 65.3	0.338	1.319 (0.748–2.326)	0.170	1.354 (0.879–2.087)
**IPI score**	0–2 *vs.* 3–5	**0.006**	88.8 *vs.* 29.3	**< 0.001**	101.2 *vs.* 62.7	**0.048**	1.945 (1.005–3.767)	0.494	1.205 (0.706–2.058)
**Hans classification**	Non-GCB *vs.* GCB	0.914	66.5 *vs.* 80.7	0.095	77.6 *vs.* 85.9				
**PD-1 on TICs**	(−) *vs.* (+)	0.797	81.6 *vs.* 76.0	0.478	85.9 *vs.* 80.7				
**PD-1 on LCs**	(−) *vs.* (+)	0.657	77.8 *vs.* 76.0	0.882	84.9 *vs.* 76.3				
**PD-L1 on TICs**	(−) *vs.* (+)	0.955	85.9 *vs.* 76.2	0.623	111.0 *vs.* 79.2				
**PD-L1 on LCs**	(−) *vs.* (+)	**0.015**	77.7 *vs.* 15.6	0.373	85.1 *vs.* 22.3	**0.034**	2.393 (1.070–5.352)		

GCB = germinal center B-cell DLBCL subtype; IPI = International Prognostic Index; LCs = lymphoma cells; N = number; non-GCB = non-germinal center B-cell like DLBCL subtype; OS = overall survival; PD-1 = programmed cell death protein 1; PD-L1 = PD-1 ligand; PFS = progression-free survival; TICs = tumor-immune cells

### Correlation of PD-1 and PD-L1 expression with progression free-survival and overall survival

**FIGURE 3.**
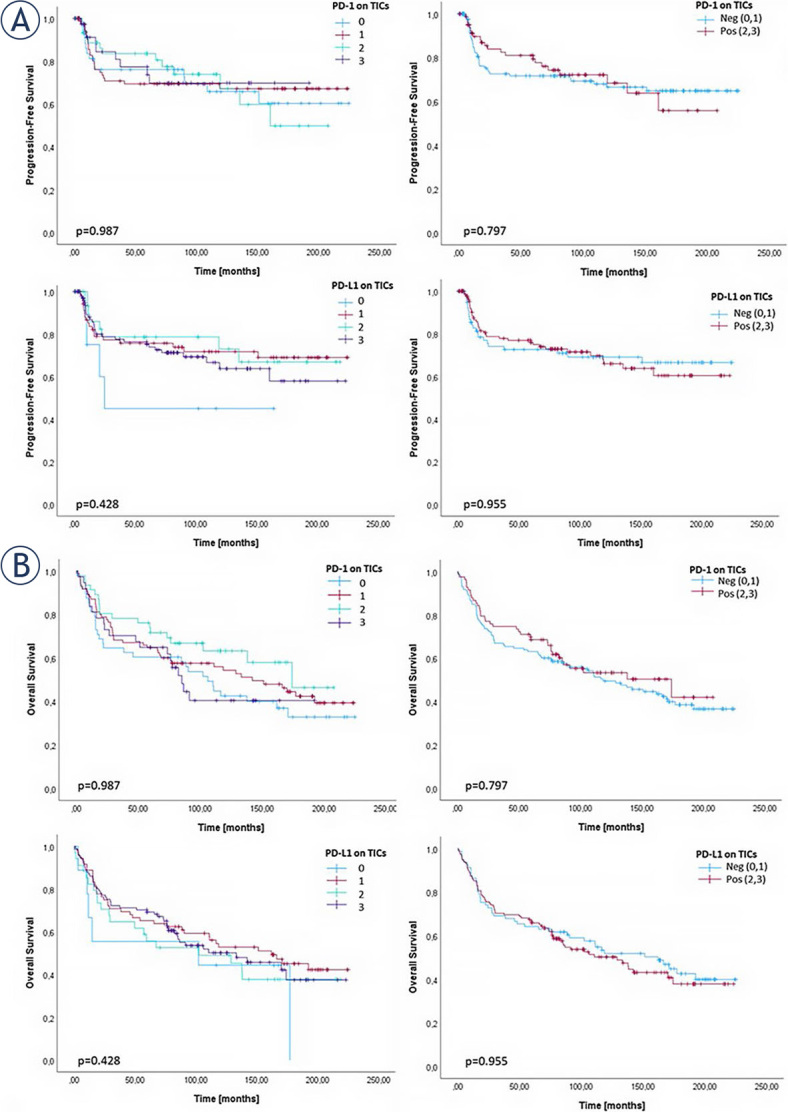
Kaplan-Meier curves for **(A)** progression-free survival (PFS) and **(B)** overall survival (OS) for PD-1 and PD-L1 on tumor-immune cells. The cases were divided into four groups based on the cell count per high-power field. Furthermore, these cases were stratified into two classifications: negative (cell counts 0 and 1) and positive (cell counts 2 and 3). PD-1 = programmed cell death protein 1; PD-L1 = PD-1 ligand; TICs = tumor immune cells.

We conducted individual survival analyses for PD-1 and PD-L1 on TICs. The results were obtained while classifying the samples into four groups, as well as grouping them in two categories: negative (0 and 1) and positive (2 and 3). However, no statistically significant differences were found in either data set (Figure 3).

**FIGURE 4.**
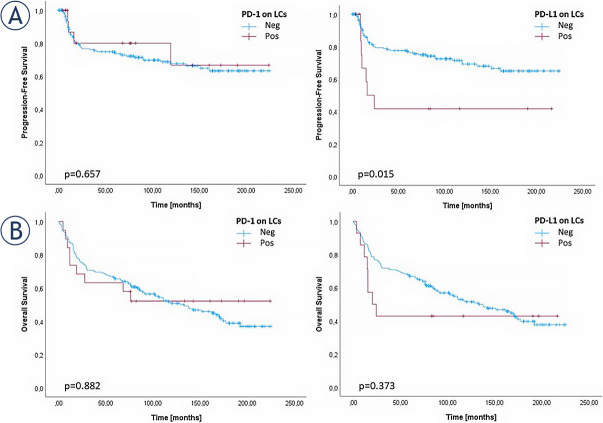
Kaplan-Meier curves for **(A)** progression-free survival and **(B)** overall survival representing the influence of PD-1 and PD-L1 expression on lymphoma cells (LCs). PD-1 expression on LCs was categorized as negative below 10%. PD-L1 expression on LCs was categorized as negative below 30%. PD-1 = programmed cell death protein 1; PD-L1 = PD-1 ligand

Correlation analysis between PD-L1 expression on LCs and patient survival showed a significant correlation for patients who had no PD-L1 expression on LCs (i.e. less than 30% of LCs were PD-L1 positive), with significantly longer progression-free survival (PFS) (p = 0.015) compared to patients who had detectable PD-L1 expression (77.7 months *vs*. 15.6 months). However, no correlation with OS was observed. Furthermore, correlation PD-1 expression on LCs cell showed no correlation with PFS and OS. Detailed results of the correlation analysis for PD-1 and PD-L1 expression with PFS and OS are summarized in Table 4 and Figure 4.

### Multivariate analysis of the significant parameters in the univariate analysis

Only the significant clinicopathological features from the univariate analysis were included in the multivariate analysis: the Ann Arbor stage, presence of B symptoms, IPI score, and PD-L1 expression on LCs were used for the PFS analysis, and the age, Ann Arbor stage, presence of B symptoms, and IPI score were used for the OS analysis. Our results showed that the IPI score (p = 0.048, HR = 1.945) and the presence of PD-L1 on the LCs (p = 0.034, HR = 2.393) retained their significant prognostic impact for PFS. As expected, patient age (p < 0.001, HR = 2.907) was found to be a significant prognostic factor for OS, while the other variables, including IPI score, remained non-significant. The results of the multivariate analysis are shown in Table 5.

## Discussion

In the present study, we investigated the expression of PD-1 and PD-L1 on LCs and TICs in the tumor microenvironment of DLCBL, NOS patients in relation to non-GCB and GCB subtypes and patients’ survival.

The expression of PD-1 and PD-L1 has drawn great attention to the impact of lymphoma treatment, particularly in aggressive lymphomas such as DLBCL, NOS, where they evade immune response and drive aggressiveness.^21^ Identification of patients for PD-1/PD-L1 immunotherapy, possibly through IHC evaluation, holds promise for better patient outcomes and further research in checkpoint inhibitor treatment. Currently, there are few ongoing clinical trials investigating the use of anti-PD-1 and anti-PD-L1 treatments in patients with relapsed or refractory DLBCL, NOS. Preliminary results in small patient cohorts show promising results for prolonged disease-free intervals.^29^ However, the selection criteria for patient enrollment are not dependent on PD-1 or PD-L1 expression on either TICs or LCs. One of the explanations for this could be the lack of consensus in the evaluation criteria, especially for PD-1 expression, as it is less frequently assessed and less defined. Different evaluation criteria are used to assess cell positivity, and we have used the most commonly used ones.^8,14,16,17,25,29,32^ We found only a few studies investigating whether there is a correlation between the expression of PD-1 or PD-L1 on TICs or LCs and patient outcomes. For the assessment of PD-1 and PD-L1 on LCs and TICs in our study, we performed double staining for PD-1/PAX5 and PD-L1/PAX5 to simplify the assessment of PD-1 and PD-L1 expression and obtain more reliable results. Indeed, PAX5 was required for accurate identification of B cells, including LCs, because PAX5 is expressed in mature B cells and LCs.^14,24,25,33^ The use of PAX5 increased the accuracy of LC identification, which in combination with the simultaneous staining of PD-1 and PD-L1 is one of the major advantages of this study. This double staining was so far reported in Kiyasu’s study of 1091 patients with DLBCL^14^, NOS and Chen’s study of various lymphoma subtypes, including 66 patients with DLBCL, NOS^33^, but both studies used only PD-L1/PAX5 staining. To the best of our knowledge, our study is the first to perform PD-L1 and PD-1 staining simultaneously with PAX5.

In this way, we confirmed a PD-1 expression of 38.4% on TICs and 8.8% on LCs. Our results were similar to the already published data, where PD-1 expression on TICs ranged from 22.2–60.0%. For PD-1 on LCs, we observed a slightly lower expression rate compared to the expression levels of PD-1 reported in the literature (22.2–65.0%).^16,24,25,29,34^ Using the same assessment method as in the other published studies^14,16,17,25,29,30^, we reported 62.4% PD-L1 expression on TICs and 6.5% on LCs. Our data again differed from already reported PD-L1 expression levels, where expression ranged from 15.3–37.0% on TICs and 8.9–61.1% on LCs.^14,17,24,29^ In summary, we observed a lower expression of PD-L1 and PD-1 on LCs and a higher expression of PD-L1 on TICs. We speculate that the major reason for the low expression on LCs is the additional staining with PAX5. With the double staining, we were able to recognize PD-1/PD-L1 positive LCs more precisely, so the numbers are probably more reliable than in other studies where other cells such as macrophages could be misinterpreted as LCs and resulted in higher expression of PD-L1 and PD-1 on LCs. Two already published studies using PAX5 staining reported 8.9%^14^ and 11% PD-L1 positivity on LCs^33^, which is comparable to our results.

We also speculate that a possible reason for this discrepancy in PD-L1 and PD-1 expression rates could be a consequence of several other factors. One reason might be the different inclusion criteria of the patients. Almost all published studies included patients with Epstein-Barr virus (EBV) positive DLBCL, NOS, which typically exhibit PD-L1 positive status due to the association between PD-L1 expression and EBV infection. EBV positive patients were not a part of our patient cohort, so this could be one of the reasons for the discrepant results. In addition, different algorithms were used to define the DLBCL, NOS subtypes. The majority of the studies have used Hans’ algorithm^14,16,17,25,29,30^, but some have applied Choi’s algorithm.^35^ The choice of one of these algorithms may contribute to different percentages of GCB and non-GCB subtypes, as well as different percentages of PD-L1 positive LCs and TICs within each subtype.^35^ Furthermore, different antibody clones were used in different studies, and in some cases the clones used were not clearly stated.^8,25^ Additionally, some studies lacked a clear description of the criteria used for the assessment of PD-L1 positivity.^24^ Since the major focus was on the Asian population, where non-GCB subtypes of DLBCL, NOS, were more common than GCB subtypes, a higher number of PD-L1 positive cases was expected.^22^

The second aim of our study was to investigate the association of PD-1 and PD-L1 expression on TICs or LCs with clinicopathological characteristics. We investigated the potential differences between various clinicopathological characteristics of patients such as age at diagnosis, gender, Ann Arbor stage, extranodal organ involvement, involvement of the spleen, presence of B symptoms, and IPI score, but our results showed no difference in PD-1 and/or PD-L1 expression on LCs and/or TICs between these groups. Additionally, we examined the histological subtypes determined by the Hans algorithm and found that PD-L1 positive expression was more frequent in the non-GCB subtype, as already reported.^14,15,17,19^ However, in a univariate analysis, the Hans algorithm showed no significant impact on PFS or OS. Regarding patient survival, we found that patients with PD-L1 expression on the LCs are correlated with shorter PFS, which also had a significant impact in the multivariate analysis including the IPI score. This indicates that although the number of patients with PD-L1 positive expression on the LCs is small, their survival is worse in comparison with the patients with no PD-L1 expression, regardless of the IPI score. Since the IPI score is still one of the strongest and most reliable prognostic markers in DLBCL, NOS, even in the era of novel therapies, we believe this is an interesting conclusion that requires further research.

In the multivariate analysis for PFS, where the Ann Arbor stage, presence of B symptoms, IPI score, and PD-L1 expression on LCs for PFS were included as significant parameters from the univariate analyses, PD-L1 expression on LCs was found to be an independent prognostic marker for PFS. As expected, the IPI score also proved to be an independent prognostic marker for PFS. In the multivariate analysis of OS, where the age, Ann Arbor stage, B symptoms and IPI score were included, we showed that age remained as an individual marker for OS. This result was consistent with already published data based on 5-year follow-up analyses for PFS and OS of DLBCL, NOS patients.^8^

Noteworthy, there are some limitations of our study that need to be considered. For example, due to the retrospective nature of the study, the longer archiving time of FFPE tissue blocks may potentially influence the staining results. In addition, it is still unclear whether the expression of PD-1 and PD-L1 on tumors and TICs is a key factor for the clinical prognosis of DLBCL, NOS patients treated with PD-1/PD-L1 blockade therapy. On the other hand, our study has certain advantages, such as centralized evaluation of specimens, homogeneous treatment and long follow-up time. To the best of our knowledge, we are the first to simultaneously assess the expression of PD-1 and PD-L1 on both TICs and LCs from the same cohort of patients using a double immunostaining approach and their impact on PFS and OS, as well as their association with other clinicopathological characteristics. Our results were consistent with individual studies on the European population as well as studies on the Asian population.

In conclusion, we demonstrated that PD-L1 expression on LCs was associated with shorter PFS and was more frequently observed in the non-GCB subtype. Double IHC staining with PAX5 proved to be a feasible method to assess PD-1 and PD-L1 expression in tissue samples. Further research and clinical studies are required to assess the importance of assessing PD-1 and PD-L1 in DLBCL, NOS patients as well as methods to determine their expression, particularly with regard to planning immunotherapy treatments. It is also important to understand the mechanisms of tumor immune evasion induced by PD-1/PD-L1 and to explore approaches to modulate the host immune response accordingly, which requires further research.
